# Rethinking the complexity and uncertainty of spatial networks applied to forest ecology

**DOI:** 10.1038/s41598-022-16485-9

**Published:** 2022-09-23

**Authors:** Hao-Ran Wu, Chen Peng, Ming Chen

**Affiliations:** Department of Bioinformatics, College of Life Sciences, Zhejiang University, Hangzhou, 310058 China

**Keywords:** Ecology, Ecology

## Abstract

Characterizing tree spatial patterns and interactions are helpful to reveal underlying processes assembling forest communities. Spatial networks, despite their complexity, are powerful to examine spatial interactions at an individual level using well-defined patterns. However, complex forestation networks introduce uncertainties. Validation methods are needed to assess whether network-based metrics can identify different processes. Here, we constructed three types of networks, which reflect various aspects of tree competition. Based on five spatial null models and 199 Monte-Carlo simulations, we were able to select network-based metrics that exhibited well performance in distinguishing different processes. This technique was then applied to a tropical forest dataset in Costa Rica. We found that the average node degree and the clustering coefficient are good metrics like the paired correlation function. In addition, the network approach can identify fine-scale spatial variations of tree competition and its underlying causes. Our analyzes also indicate that a bit of caution is needed when defining the network structure as well as designing network-based metrics. We suggested that validation techniques using corresponding spatial null models are critically important to reduce the negative effects caused by uncertainties of the network.

## Introduction

Forest plays an important role in the global carbon cycle^1,2^, biodiversity maintenance^3^, and human well-being^4^. Despite the critical values of forests to help mitigate human-caused climate change, climate-driven risks^5^, as well as habitat fragmentation^6,7^ have posed a threat to forest stability. Due to the rapid deforestation in the past decades, forest restoration has been regarded as a priority on a global scale^8^. Clear understanding and quantification of mechanisms assembling forest communities are likely to have substantial benefits for forest restoration and biological conservation^9–11^.

Various processes—ranging from environmental filtering, dispersal limitation, and competition to disturbance—have an impact on forest structure^12^. Successful colonization of a particular site relies on the capacity to overcome environmental and biotic barriers^13^. The latter includes species interactions like competition and the Janzen–Connell (JC) effects^14,15^, which are closely related to the structure and dynamics of the forest community^16^. Inter-tree competition for resources (i.e. light, space, and nutrients) may be prevalent^16,17^. However, due to its spatial variability, processes altering tree interactions are still poorly understood. One of the mechanisms that increase density-dependent tree mortality is dispersal limitation^18^. Seeds that fail to reach remote sites may undergo a higher level of competition^19^. Other processes like fire exclusion^20^, wind disturbance^21^, and flooding^22^ may reduce tree competition, thus altering the mortality patterns and spatial arrangement of tree interactions. Studies have shown that higher tree densities did not necessarily translate into increased mortality^23^. In the seedling stage, facilitation across individuals is prevalent. Strong competition is usually detected in adult trees^17,24,25^. Closed phylogenetic relationships can also lead to a higher level of competition^25^. Thus, techniques are needed to decompose spatial variations of tree interactions and reveal their underlying mechanisms.

During the past decades, interest in methods for analyzing spatial datasets expanded rapidly in ecology^26–29^. It is critical for evaluating ecological theories^30^. Existing spatially explicit metrics applied to community ecology range from spatial point pattern analysis and quadrat-based analyses to individual-based neighborhood models, which are powerful to reveal spatial processes in fully mapped communities at several scales simultaneously^26^. For spatial variations of tree interactions, the limitation of such analyses lies in the assumption that tree competes for space, regardless of light and nutrients. Also, local spatial variations are usually ignored in these metrics. For example, many summary statistics derived from Ripley’s *K* statistic^31^ count for averaged spatial crowdedness within a specific region. To quantify clumping, these measures implicitly assume homogeneous intensity of point process^19,27^. Nearest-neighbor statistics^26,32–34^ identify spatial clusters using nearest distance approaches. Despite its powerfulness, many ecological processes, such as habitat filtering, can be better revealed by identifying the spatial position of each cluster^30^.

Ecological networks, despite their complexity (i.e. various definitions with lots of variables to be considered), are powerful to reveal ecological mechanisms using well-defined patterns^35–37^. Network systems usually include nodes (i.e. units of biological hierarchy) and edges (i.e. interactions between nodes), which defines the network structure of ecological systems rather than pairwise interactions^38^. Mathematical approaches, including the analysis of graph-theoretical properties, can be applied to examine interactions among large numbers of nodes efficiently^38^. For plant ecology, networks are used to characterize individual-level interactions such as competition, facilitation, and predation^16,35,36,39^. By introducing binary and weighted out-degrees, Nakagawa et al.^39^ found that larger plants competed more strongly with other large plants in 1948, but they competed preferentially with small plants after 30 years in Hokkaido, Japan. By constructing forestation networks, Schmid et al.^16^ found that pioneer species did not tend to be shaded by other trees. Despite its powerfulness to reveal additional ecological information, complex tree competition networks introduce uncertainties. Attempts should be made to validate the relatedness between network-based spatial explicit metrics and underlying processes.

**Figure 1:**
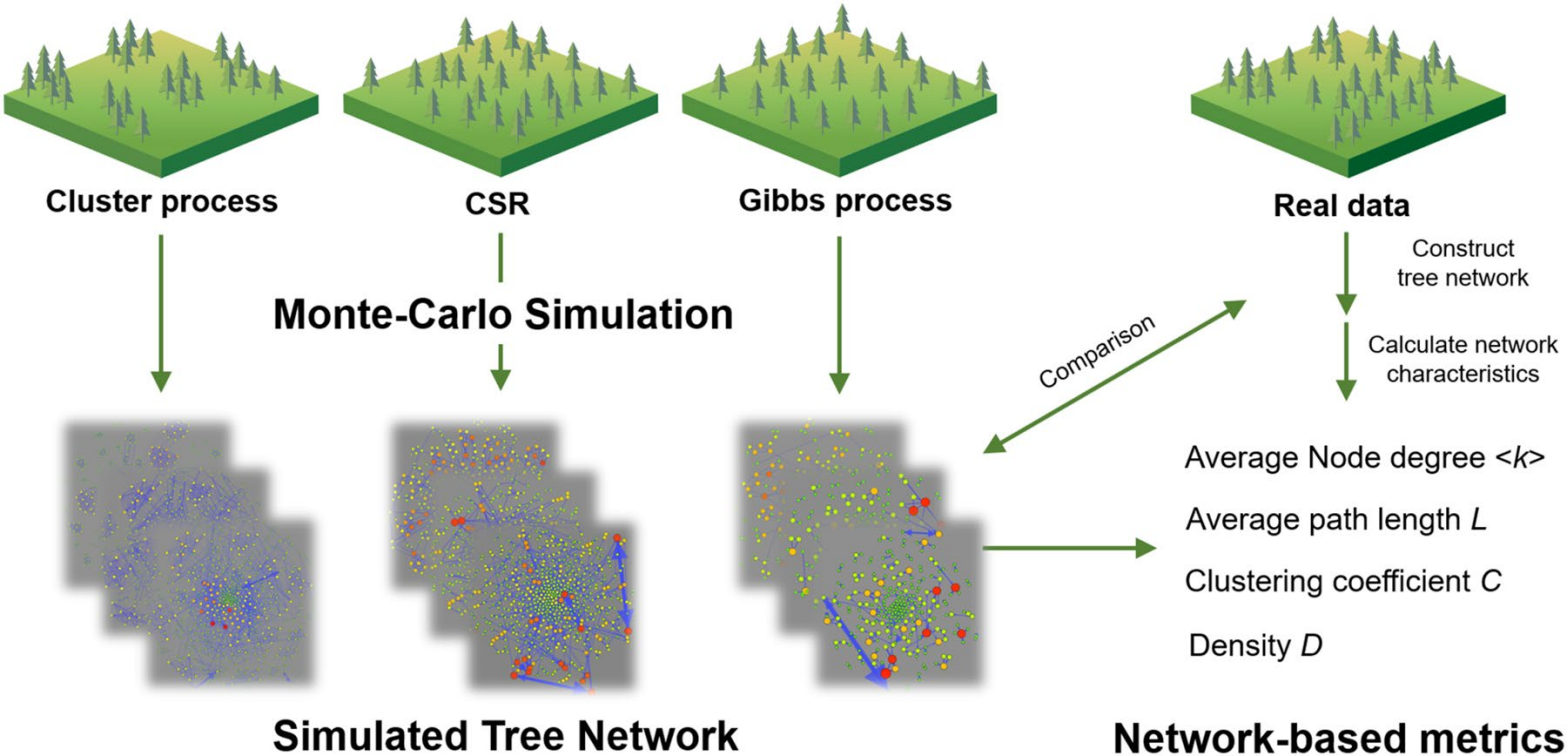
Overview of the procedure using network characteristics to examine spatial patterns of tree competition. Monte-Carlo simulations of various spatial null models should be carried out to translate ecological processes into corresponding patterns. Network-based metrics are then calculated for each realization of the simulation. Metrics that fail to distinguish different processes should be discarded. The last step is to perform network analysis based on empirical data and make comparisons between empirical and simulated results.

In this article, we develop an approach to characterize the spatial distribution of individual-level tree interactions based on network approaches (Fig. 1). We construct three types of networks (with individual trees as nodes) to characterize tree competition for light and space: (1) Competition for space (CS), where trees are connected within a fixed distance; (2) Competition for light (CL), where trees with overlapping crowns are connected; (3) Weighted competition for light (WCL), which is the same as CL except that the intensity of interaction is weighted. Using several spatial null models (i.e. complete spatial randomness (CSR), which generates random distribution patterns; Matérn process, which generates aggregated patterns; Thomas process, which generates aggregated patterns; Gibbs hard core process (HC), which generates regular patterns; Strauss process, which generates regular patterns) with different ecological hypotheses, we validate network-based spatially explicit metrics (i.e. average node degree, average path length, density, clustering coefficient) by Monte-Carlo simulations. For each spatially explicit metric, ranging from network-based metric to Ripley’s *K* and pair correlation function (PCF) function *g*(*r*), we compare the ability of each metric to characterize tree spatial patterns and interactions, and the ability to reveal underlying processes. To test the effectiveness of our network-based metrics, we further applied this technique to some plots (1 ha) tropical rainforest dataset in La Selva Biological Station (Costa Rica) to investigate the intensity and spatial distribution of tree competition. We conclude by discussing the advantages of the network approach applied to spatial ecology as well as some cautions that need to be considered for the design and validation of the spatially explicit metrics in general.

## Results

### Network characteristics of tree competition

**Figure 2:**
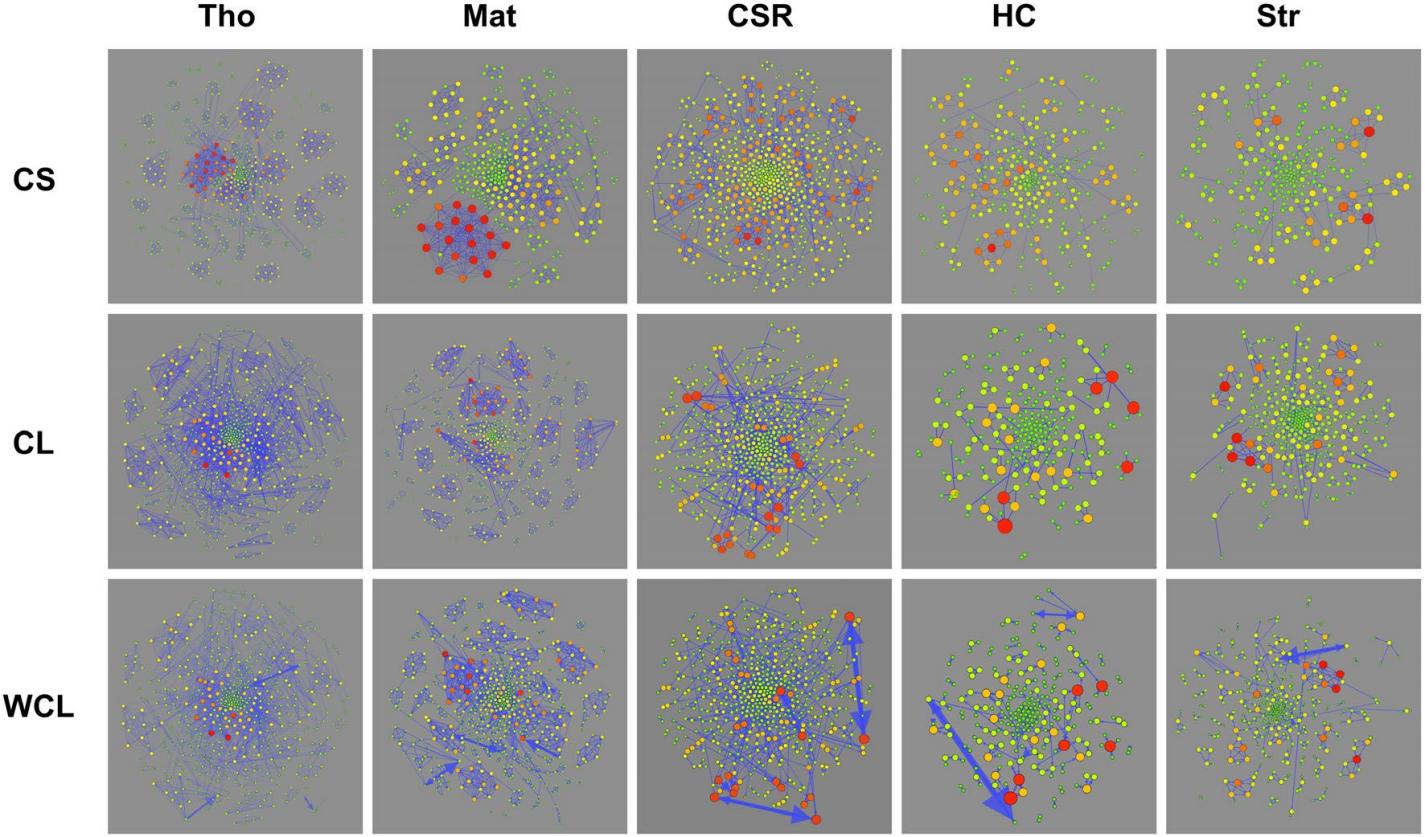
Three types of networks (*CS* competition for space, *CL* competition for light, *WCL* weighted competition for light) for each spatial null model. Each node represents a tree, and each edge reflects competition. The size and color of the node reflect the degree of the node, the redder nodes have a greater degree. In WCL, the weight is proportional to the size of the edges. Arrow size reflects the intensity of asymmetric competition. The networks are drawn using Gephi 0.9.2 with a layout of ForceAtlas2.

For each spatial null model, we constructed three types of networks (Fig. 2). 199 Monte-Carlo simulations were performed for each type of null model (see Supplementary Fig. S1 for distributions of trees). A total of 1990 undirected forestation networks (CS and CL) and 995 bi-directed forestation networks (WCL) were constructed. We found that forestation networks based on cluster models were highly connected. The Thomas process always promotes the highest number of edges on average. The Matérn process promotes the second-highest number of edges on average. For CSR, the number of edges is considerably lower than that produced by Thomas and Matérn processes. The Strauss process produces the second-lowest number of edges on average, followed by the HC process (see Supplementary Table S1 for details).

**Figure 3:**
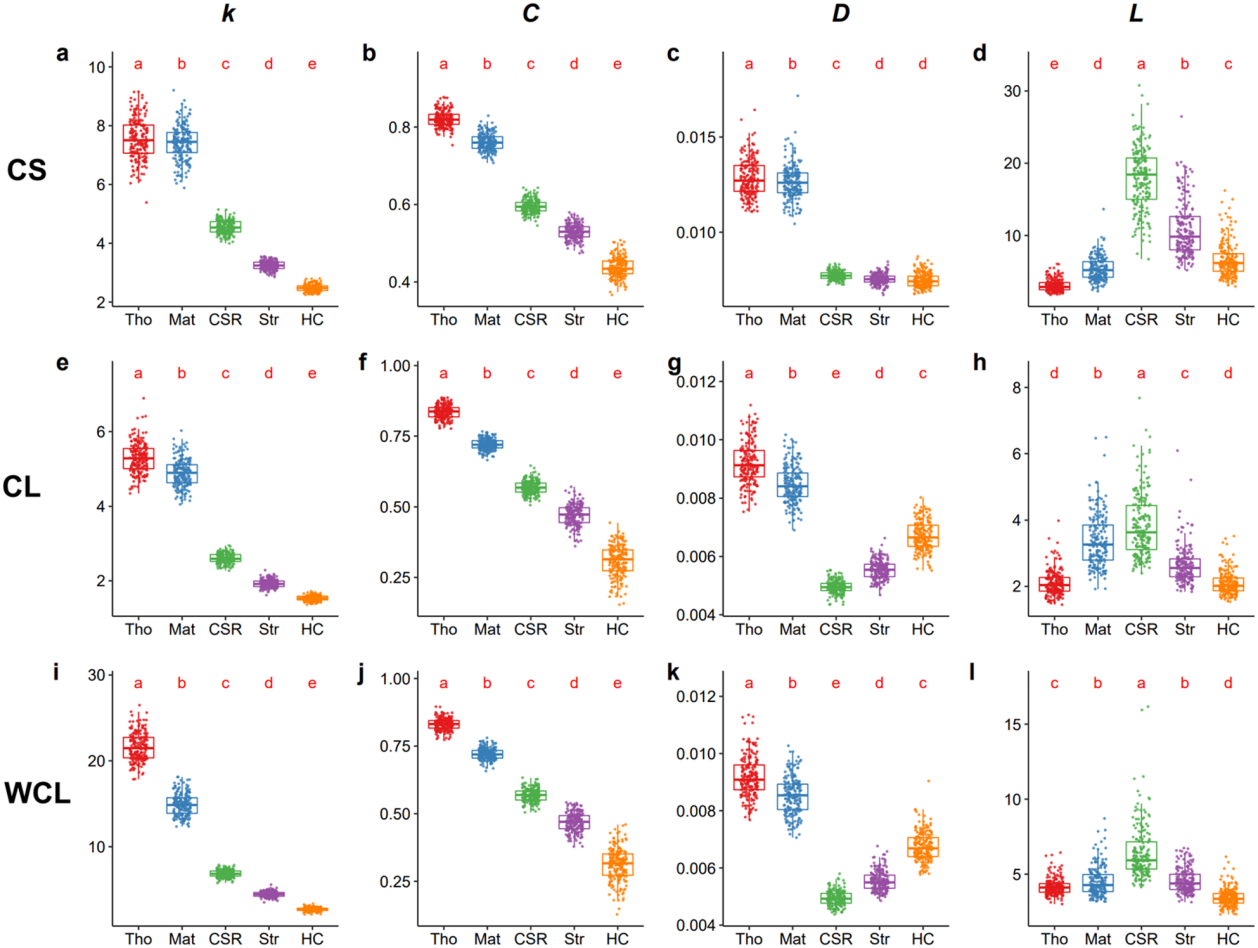
Basic characteristics of three types of networks (with individual trees as nodes) based on five spatial null models (*CSR* complete spatial randomness, *Mat* Matérn process, *Tho* Thomas process, *HC* Gibbs hard core process, *Str* Strauss process). Values of each metric (the average node degree *k,* the clustering coefficient *C*, the density *D*, and the average path length *L*) are generated by 199 Monte-Carlo simulations. Differences among five models were examined by ANOVA, followed by Tukey multiple comparisons tests (for more details, see Supplementary Table S2). In WCL, the weighted average node degree *k* was calculated, while values of *C*, *D*, and *L* are consistent with that of CL because edge weights are not used when calculating these metrics.

Both average node degree *k* and clustering coefficient *C* showed a distinctive difference among five processes (Fig. 3; See Supplementary Table S1 for details). Examining four network-based across three types of networks, the Thomas process exhibited the highest *k* values, slightly greater than that produced by the Matérn process. The CSR process exhibited considerably lower *k* values, followed by the Strauss and finally the HC processes. No overlapping ranges of *k* value among cluster processes, CSR, and Gibbs processes were detected (Fig. 3a,e,i). The distributions of *C* values showed similarities to *k* values except that the difference between the values produced by the Thomas and Matérn process was greater. The sensitivity test showed that variations in parameters could not alter these trends (see Supplementary Figs. S2–S5 for details).

The density *D* and the average path length *L* of the network, however, failed to distinguish different processes. In CS, for instance, no significant differences were found between *D* values generated by CSR and Gibbs processes (Fig. 3c). Overlapping of *D* values among various models was frequent in three types of networks (Fig. 3c,g,k). For *L* value, huge variance and many outliers within each model were found, leading to its poor ability to distinguish different types of the models (Fig. 3). When varying model parameters, these two metrics changed dramatically (see Supplementary Figs. S2–S5 for details). For instance, when *r*_*d*_ = 3 m, the HC process produces a lower *D* compared to the cluster processes in CL and WCL. However, the *D* value generated by HC rose considerably and became higher than that produced by the cluster processes when *r*_*d*_ = 6 m (Supplementary Fig. S4g,k).

**Figure 4:**
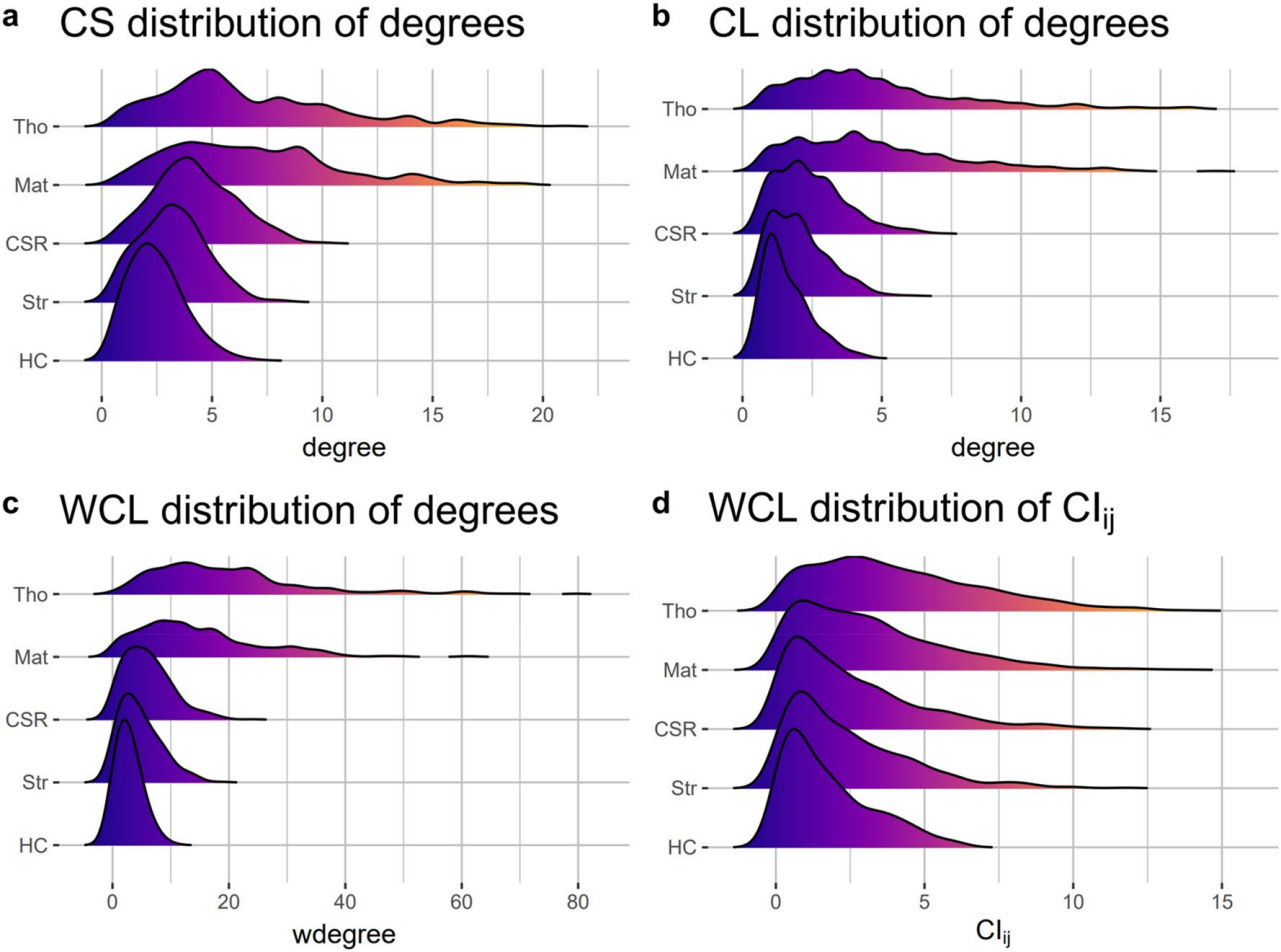
Node degree distribution and edge distribution of the network (with individual trees as nodes). (**a**,**b**) The node degree distribution of CS and CL networks based on five spatial null models. (**c**) The weighted degree distribution of WCL. (**d**) Distribution of edge weight (namely CI) in WCL.

The results of node degree distribution in various models are shown in Fig. 4. In CS, most of the nodes suffered competition from less than five neighbors in Gibbs processes, while in cluster processes some points suffered competition from an extremely great number of neighbors (Fig. 4a). A similar pattern was found in other types of networks (Fig. 3b,c). For cluster processes, variation of node degree was significantly larger than that in CSR and Gibbs processes. The peak value of probability density in cluster models was much lower. On the contrary, no significant differences were found for edge weight distribution in WCL (Fig. 4d).

### Spatial patterns of tree interactions

**Figure 5:**
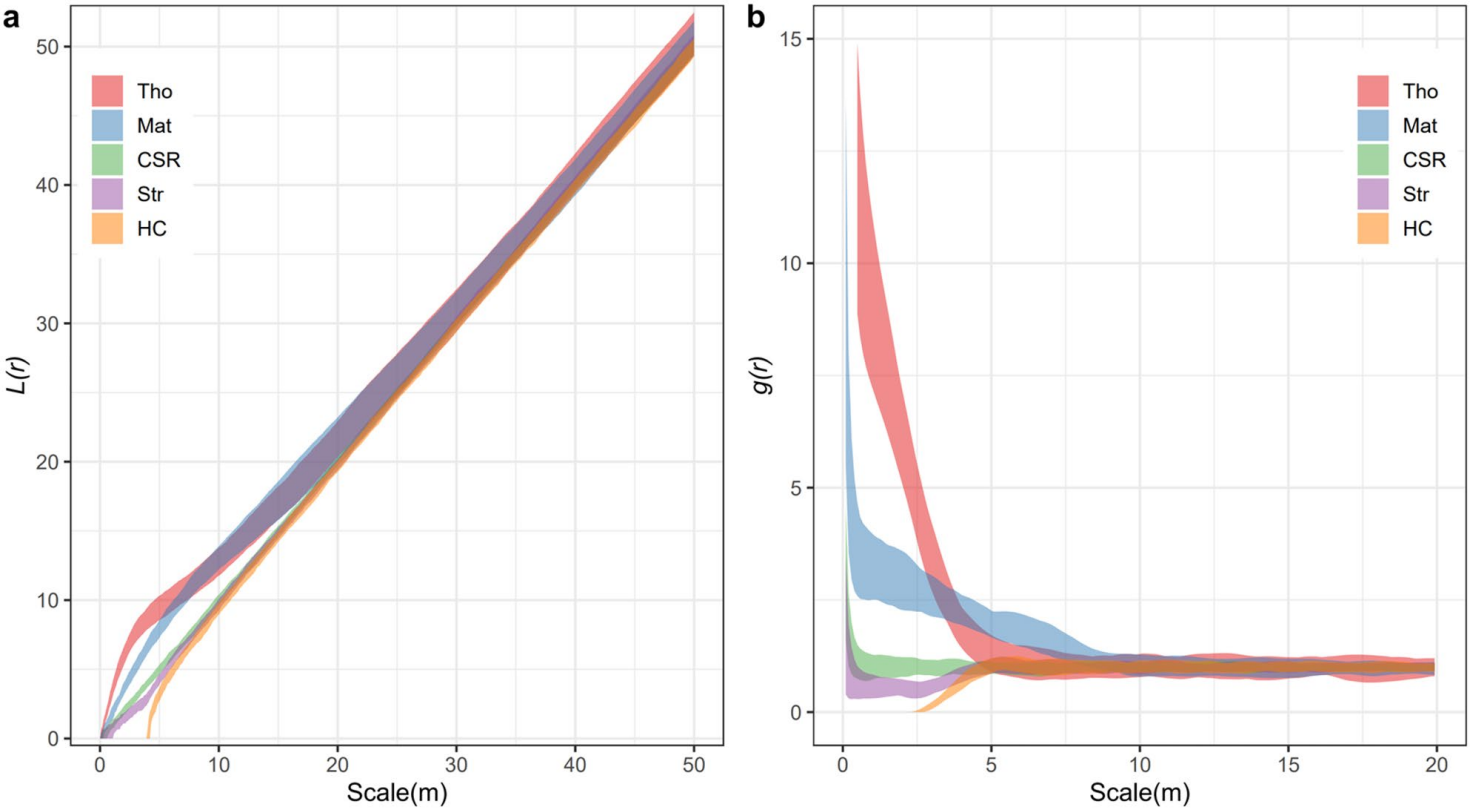
Variations of univariate *L*(*r*) and *g*(*r*) functions based on five spatial null models generated by 199 Monte-Carlo simulations. (**a**) *L*(*r*) function. (**b**) *g*(*r*) function. The boundary of envelopes reflects the maximum and minimum values of *L*(*r*) and *g*(*r*) functions in the simulations. The colorful areas show the 95% confidence limits around the predicted *L*(*r*) or *g*(*r*) estimated from 199 random simulations of the point process.

Based on 199 Monte-Carlo simulations, the univariate *L*(*r*) function showed different patterns among various spatial null models (Fig. 5). At scales *r* < 15 m, the lower bound of the envelope generated by the Thomas process was higher than the upper bound of the envelope by CSR. Similarly, the envelope generated by CSR exhibited higher values than that by HC at short distances, *r* < 10 m. When the scales become larger, overlaps were detected between different envelopes. Envelopes calculated by *g*(*r*) showed similarities to that by *L*(*r*). At scales *r* < 5 m, the envelope by Thomas process showed the highest values, followed by CSR and HC. Again, overlaps occurred when scales became larger.

### Spatial variations of network characteristics

We found that average node degree *k* showed distinctive distribution patterns among different spatial models (Supplementary Fig. S6). Cluster processes exhibit high levels of spatial inhomogeneity. The spatial distributions of *k* value in Gibbs processes showed a smooth trend with low values in most regions. Betweenness centrality in CSR was generally much higher compared to that in cluster and Gibbs processes.

### Application of network-based metrics to tropical rainforest dataset

**Figure 6:**
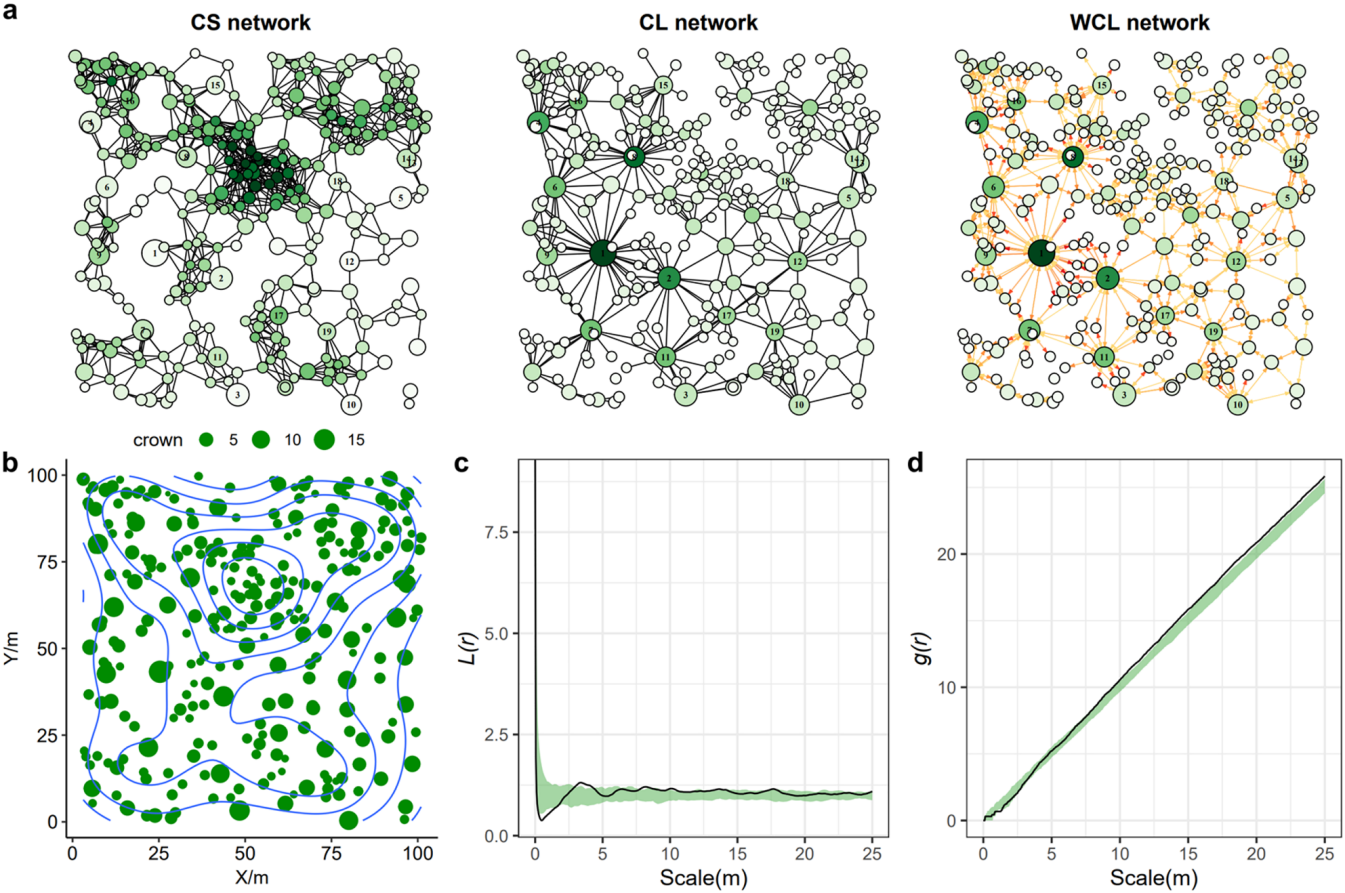
(**a**) Three types of networks (*CS* competition for space, *CL* competition for light, *WCL* weighted competition for light) based on the forested swamp plot FS-1 in Costa Rica. Each node represents a tree, and each edge reflects competition. The size of the node reflects the radius of the tree crown. Deeper colors indicate a higher value of node degree. In WCL, the weight is proportional to the width and color depth of the edges. The nodes in the networks retain spatial coordinates in panel (**b**). (**b**) Distribution of trees in the plot. The size of the node reflects the radius of the tree crown. The blue contour indicates the density of trees. (**c**,**d**) Variations of univariate *L*(*r*) and *g*(*r*) functions based on the CSR model generated by 199 Monte-Carlo simulations. The boundary of envelopes reflects the maximum and minimum values of *L*(*r*) and *g*(*r*) functions in the simulations. The black line represents observed values estimated on the tropical rainforest dataset.

**Figure 7:**
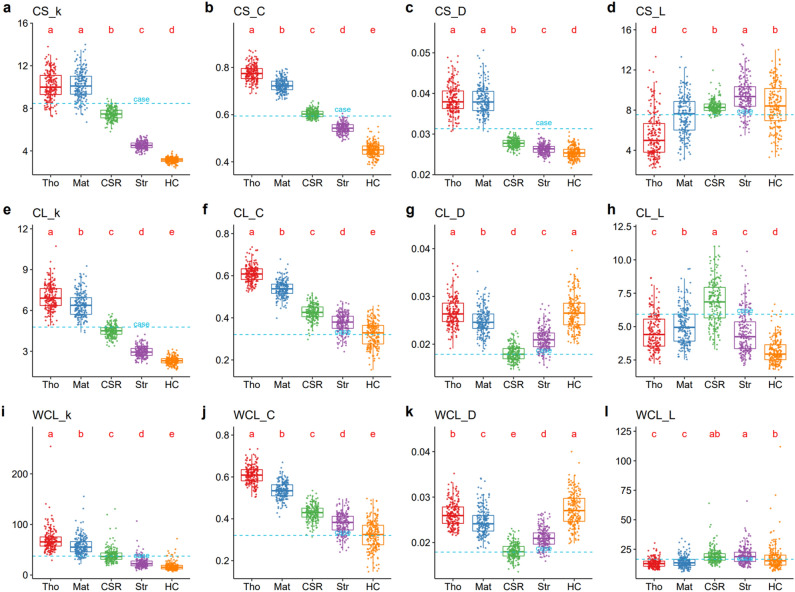
The basic characteristics of three types of networks (with individual trees as nodes) estimated on the forested swamp plot FS-1 in Costa Rica (represented by the horizontal blue line). Simulated metrics are generated based on five spatial null models (*CSR* complete spatial randomness, *Mat* Matérn process, *Tho* Thomas process, *HC* Gibbs hard core process, *Str* Strauss process) with parameters estimated on the dataset to be comparable. Values of each metric (the average node degree *k,* the clustering coefficient *C*, the density *D*, and the average path length *L*) are generated by 199 Monte-Carlo simulations. Differences among five models were examined by ANOVA, followed by Tukey multiple comparisons tests (for more details, see Supplementary Table S2). In WCL, the weighted average node degree *k* was calculated, while values of *C*, *D*, and *L* are consistent with that of CL because edge weights are not used when calculating these metrics.

We calculated four network-based metrics for the tropical rainforest plot in Costa Rica, which are listed in Table 1. Locations, where trees suffer from high levels of competition in the CS network, are different from that in CL and WCL networks, indicating that large tree crowns may alter interaction patterns considerably (Fig. 6a). Based on the results of *L*(*r*) and *g*(*r*), we found random patterns for almost all scales (Fig. 6c,d), which coincided with the results given by the clustering coefficient *C* in the CS network. The average node degree *k* falls in the transition zone between CSR and cluster processes in the CS network (Fig. 7a). However, when adding information about tree crown into the network, the competition strength becomes lower. Although the value of averaged node degree *k* remains in the region of CSR, the result of clustering coefficient *C* exhibits regular patterns. Although the values density *D* and the average path length *L* fall in the region of CSR, there are many overlaps between the simulated values of different processes, making it not sufficient to identify a process with high levels of confidence (Fig. 7).

**Table 1:** Network metrics of the forested swamp plot. Indices in the table includes: *N* the number of nodes, *E* the number of edges, *k* the average node degree, *D* the density of the network, *L* the average path length. The value of *C*, *D*, *L* in WCL is identical to CL due to the same definition of network except for edge weight.

	*N*	*E*	*k*	*C*	*D*	*L*
CS	271	1146	8.458	0.594	0.031	7.546
CL	268	640	4.776	0.321	0.018	5.928
WCL	268	1280	37.445	0.321	0.018	16.536

## Discussion

Both node degree and clustering coefficient seem to be good spatial explicit metrics to examine tree spatial patterns. We found that average node degree *k* showed a distinctive pattern without any overlap ranges among cluster processes, CSR, and Gibbs processes. Average clustering coefficient *C* also showed its ability to characterize different processes in specific ranges despite some minor overlaps (Fig. 3). According to the definitions of the cluster processes, a set of ‘parent’ points is randomly generated before each of them gives rise to a random pattern of ‘offspring’ points within a disc and dies^28,40^. Nodes in the networks are hence more likely to find close neighbors within a short distance, leading to a higher node degree. Clustering coefficient *C* analyzed the local density of the network. ‘Offspring’ points generated by cluster models tend to be closer compared to points generated by CSR, thus the division of the number of connected edges by maximum possible edges within a local region is higher.

Despite their adequate performance in distinguishing various processes, average node degree *k* and cluster coefficient *C* showed different levels of variability. For cluster models, a huge variation in average node degree was found (Fig. 3a,e,i). One of the reasons might be variations in the paired distance between clusters. Since ‘parent’ points are generated according to CSR, the clusters are randomly distributed. Isolated clusters can lead to low values of *k*. However, the clustering coefficient calculates the number of connected edges between the neighbors of nodes^16^, thus diminishing the effects of cluster distance. We also found little variations of *k* value in Gibbs models (Fig. 3a,e,i). In Gibbs processes, strong interactions (represented by closed distance) between trees are not allowed. Hence a uniformly distributed pattern would be generated^28^, which might reduce the variations of the number of neighbors. Consequently, we advised that average node degree *k* is plausible to identify regular patterns while cluster coefficient *C* is a good metric to examine aggregated patterns.

Node degree distributions of the networks followed heavy-tailed distributions, similar to a wide range of previous studies in forest ecology based on network approaches^16,36,39^. Particularly, node degrees in cluster models were highly variable (Fig. 4a–c), indicating that some trees suffer competition from a high number of neighbors, while others suffer competition from a few neighbors. Trees in isolated clusters tend to have fewer neighbors, while trees in clusters that are closed to each other may have more neighbors. For CSR and Gibbs processes, the distributions are narrower, indicating reduced competitions in a random or regular pattern.

Unfortunately, the density *D* and the average path length *L* of the network fail to distinguish the spatial models. The average path length *L* is a measure of global connectivity or spatial segregation of the network^16,36,41^. It calculates the shortest distance between two connected nodes, quantified by the number of edges between them^36^. According to the definition of CL and CS networks, the path of a pair of points exists if there are enough neighbors or overlapping tree crowns between them, which introduces uncertainties when calculating the *L* value. A similar mechanism might also exist for density *D*. Such uncertainties can be seen when varying different model parameters, which leads to irregular changes in *D* and *L* (see Supplementary Fig. S2–S5).

For the real forest dataset, we showed how network-based metrics can be used to reveal underlying processes. Based on the CS network, we found that the CSR process can explain the interaction patterns assuming that trees only compete for space (Fig. 7a,b). This result coincides with that of *L*(*r*) and *g*(*r*) (Fig. 6c,d). This pattern is also found in many studies in the natural forest^20,42^. However, a random pattern does not necessarily mean a lack of interactions, because it might come from the diversification of ecological processes^43^, invoking nothing more than the central limit theorem applied to multiple factors^44^. Our results show that considerations of the tree crown in the network turn the interaction pattern to a more regular one (Fig. 7e,f,i,j). Trees with a large crown but fewer neighbors may suffer a higher level of competition compared to trees with a small crown but more neighbors, as indicated by the difference between CS and CL networks (Fig. 6a). Therefore, observed interaction patterns might be completely different with regard to light, nutrients, and other aspects, which is a caution in research of spatial ecology.

Network-based metrics are a powerful way to analyze complex interactions in forests^16^. Like Ripley’s *K* and *g*(*r*) (Fig. 5), we found that network characteristics such as average node degree *k* and cluster coefficient *C* exhibited adequate performance in examining underlying processes, at least at small scales (Fig. 3). In the CS network, average node degree *k* is closely related to Ripley’s *K*, which is exactly the value of *k* divided by the density of trees in a region. However, networks can have many definitions like CL and WCL, which helps to characterize interactions mechanistically. In addition, various network-based metrics and analyzing techniques are available to examine fine-scale structures of interations^35^. Based on spline interpolation, attempts can be made to infer the regions with high competition values. Betweenness centrality has been applied in previous studies to identify the populations that have a huge impact on gene flow^45^. In this study, we found regions that largely affected the overall structure of competition networks indicated by high values of betweenness centrality (Fig. 6b and Supplementary Fig. S1). For forest management, selective loggings on trees with high centrality scores can be made to reduce competition and promote seed survival.

Nevertheless, a bit of caution is needed when designing the network-based spatial explicit metrics. Ecology networks are highly complex, with their general patterns and underlying causes still debated^37^. Network and graph theory provide a flexible conceptual model that can help identify the relationship between network structures and processes^46^. Information like competition kernel functions was added to the networks in the previous studies^39^. We cautioned that, however, using complicated networks with a great number of network-based metrics may produce results with greater uncertainty and thus reducing its ability to identify underlying processes. For instance, density *D* is a good metric to identify aggregated patterns in the CS network. However, overlaps occur between the Matérn and HC processes in the CL network (Fig. 3c,g). For the average path length *L*, overlaps exist across all models and all network types (Fig. 3d,h,j). When varying model parameters such as *r*_*g*_, the values of *D* and *L* generated by the HC process are sometimes lower than that by the Thomas process. However, at other times the former becomes higher, especially in the CL network (Supplementary Fig. S4). Such uncertainties indicate their poor ability to identify the underlying processes, and adding information about tree crown into the network makes it even worse. Thus, conflicts occur in applying network theory in ecological research: (1) using complicated network definitions but uncertainties are introduced by adding new variables; (2) applying various network-based metrics (including network properties) but some of them are inefficient in identifying underlying processes.

Spatial network is a powerful tool to reveal ecological processes^16,35,36^. Network approaches can examine the fine-scale spatial distribution, connectivity, and intensity of tree interactions using a combination of geographic and crown size datasets. Networks can have many definitions and properties. It allows researchers to describe individual-level interactions based on sets of rules about when interaction occurs and how strong the interaction is. This makes it possible to explore various interaction types, ranging from competition for light, space, and nutrients, to even facilitation, predation, parasitism, etc^37^. It can include more information compared to traditional approaches such as Ripley’s *K* and the nearest-neighborhood approach. Thus, networks with different definitions and different network properties are frequently used in ecological research to characterize the patterns of interaction^35–37^. However, the flexibility of the network may also cause problems—it is not sure whether network-based metrics capture the actual interaction patterns in the ecosystem. Unfortunately, validation of these metrics was often ignored in previous studies. This paper examines the performance of three networks in identifying different ecological processes. Our results indicate that networks with complicated definitions may introduce uncertainties and then mask the actual patterns, reducing the capability of the network-based metrics to distinguish different processes. At the same time, we also showed that not every network-based metric is good at distinguishing different processes, which is another caveat to ecological research that applies the network theory. We suggest that validations of network-based metrics are of vital importance to ensure that the network reveals underlying processes. For characterizing tree spatial patterns and interactions, we advise a workflow (Fig. 1): (1) Collect inventory or remote sensing data. For the UAV-LiDAR system, for instance, software can be used to extract forest information quickly^47^. For ground investigations, researchers can use portal LiDAR devices like cellphones to get information like diameter at breast height efficiently^48^; (2) Construct the network and define the weight; (3) Validate the network-based metrics using spatial null models; (5) If the metrics can distinguish different ecological processes, then they can be applied to the research.

Overall, the current study described the tree spatial patterns and interactions using three types of networks, namely competition for space (CS), competition for light (CL), and weighted competition for light (WCL). We applied four types of network-based metrics to detect underlying ecological processes using five hypothetical models: (1) CSR, (2) Matérn process, (3) Thomas process, (4) HC, and (5) Strauss process. We concluded that: (1) Both node degree distribution and clustering coefficient are good metrics in CS and CL networks to distinguish multiple processes, (2) Network approaches are powerful tools to describe fine-scale spatial variations of tree interactions. They can also help identify units that have a large impact on the overall structure of the network and thus give management implications, and (3) Networks can have many definitions and properties, but an important precaution is a careful validation using corresponding spatial null models.

## Methods

### Dataset collection

We selected a tropical rainforest dataset from airborne LiDAR collected in 2009 using an Optech ALTM 3100 (Teledyne Optech, Toronto, Canada) scanning device. The dataset provides an exhaustive tree inventory (field and LiDAR) with forest mensuration and spatial location carried out in 148 1 ha forest plots located in the La Selva Biological Station, Costa Rica^49^. These plots were distributed over old-growth, selectively-logged, secondary, and swamp forests. Forest mensuration includes diameters at breast height, the total height of the tree, crown depth, crown area, crown volume, etc. A total of 45,360 trees were mapped in the dataset. We chose a single forested swamp plot (labeled FS-1 in the dataset) to construct the network, which included 271 trees. The spatial distribution of trees is shown in Fig. 6b.

### Construction of network

To characterize spatial patterns of individual-level competitions, all trees present within the forest plot were regarded as nodes^16^. We then defined three types of networks: (1) Competition for space (CS). In this network, trees were connected if the pairwise distance was less than 10 m, which was roughly an empirical extent of tree interactions mentioned in the previous studies^50–52^; (2) Competition for light (CL). Trees in this network were connected if they were overlapped. We used the tree crown model proposed by Wang et al.^53^ to characterize tree crown competition and define overlapping trees. In this model, a tree crown was considered as a disk (called “buffer region”) with radii *R*_*i*_ and treetop points *P*_*i*_. Two tree crowns, Tree-1, and Tree-2 with treetop points *P*_*1*_ and *P*_*2*_ were separated if the geographic distance between two treetop points is large than the sum of radii of Tree-1 and Tree-2 (i.e. ||*P*_*1*_ − *P*_*2*_|| > *R*_*1*_ + *R*_*2*_). On the contrary, trees were overlapped if ||*P*_*1*_ − *P*_*2*_|| < *R*_*1*_ + *R*_*2*_; (3) Weighted competition for light (WCL). The definition of overlapping trees was consistent with CL. In WCL, however, each edge was assigned by a value, which indicated the intensity of tree crown competition. In the case of two individual trees, we assumed that the intensity of competition for light was proportional to the crown sizes as

1 $$CI_{ij} = R_{ij} \frac{{R_{i} }}{{R_{j} }}$$

where *CI*_*ij*_ is the competition index from the overlapped *i*-th to *j*-th tree crown; *R*_*i*_ and *R*_*j*_ are the radii of the “buffer” regions of *i*-th and *j*-th tree crowns; *R*_*ij*_ is the line segment between *P*_*1*_ and *P*_*2*_ falling in “buffer” regions of both trees, which computed as^53^

2 $$R_{ij} = \left( {R_{i} + R_{j} } \right) - \left\| {P_{i} - P_{j} } \right\|$$

Obviously, the competition effect caused by *i*-th to *j*-th tree (*CI*_*ij*_) was not equal to that caused by *j*-th to *i*-th tree (*CI*_*ji*_). Hence WCL was a directed and weighted network while CS and CL were undirected networks.

### Spatial null model generation

To validate three types of networks, we used five spatial null models based on different ecological hypotheses (Fig. 1): (1) Complete spatial randomness (CSR), where the number of trees falling in any region has a Poisson distribution. Trees are independent of each other; (2) Matérn process^54,55^. It first generates parent trees with intensity *κ*, and then each parent tree gives rise to a Poisson number (*μ*) of offspring, independently distributed in a circular area of radius *r*_*d*_ centered at the parent^28,40^. Offspring are uniformly distributed in the disc (i.e. parents have a uniform dispersal kernel); (3) Thomas process^56^. It is the same as the Matérn process except that parents have a Gaussian dispersal kernel; (4) Gibbs hard core (HC) process^57^. This process is simulated by trial and error. Based on a suitable initial pattern, points are removed at random and replaced only by points that are not closer than the hard core distance *r*_*g*_ to each other; (5) Strauss process^58^. It is similar to HC, but there is a certain probability *p* that the point is retained, even if it is within the hard core distance of another point. As processes (2–3) simulate dispersal limitation hypothesis and form clusters, they are known as cluster processes, while processes (4–5) simulate competition exclusion hypothesis and are classified as Gibbs processes. In CSR, the ecological process is assumed to be random. For each process, we designated a 200 m × 200 m survey area for model generation. Then we applied one of the processes to generate tree points in the area. The next step is to apply random the labeling (RL) approach to assign the radius of tree crown *R*_*i*_ (i.e. *R*_*i*_ was assigned by random values ranging from 2 to 5 m). Finally, we constructed the networks based on the simulated data and calculated network metrics. We performed 199 Monte Carlo independent simulations for each process. For clustering processes, the average number of the offspring for each parent *μ* and the radius of dispersal area *r*_*d*_ were set as 3 and 5 m, respectively. For Strauss processes, we set *p* as 0.5, and *r*_*g*_ as 4 m. The intensity of points *κ* in all models was set as 0.015. We tested the sensitivity of our models to changes in values of the parameters *κ*, *r*_*d*_, *r*_*g*_, and *μ* to examine how network-based metrics change. For the real forest dataset in Costa Rica, we also used the above five null models to generate network-based metrics, making it comparable to metrics estimated by the real dataset. The survey area was assigned to 100 × 100 m, consist with the area of the real dataset. Parameters *κ* was estimated from the density of trees in the real dataset. The crown radius *R*_*i*_ was calculated from the crown area *A*_*i*_ ($A_{i} = \pi R_{i}^{2}$). The distribution of crown radius was fitted with the gamma distribution model (see Supplementary Fig. S7 for details).

### Tree spatial patterns and interactions characterization

To explore spatial patterns of tree interactions, we used several spatial explicit metrics (Fig. 1). For network analyses, we computed four types of network characteristics, namely the density *D* of the network, the average node degree *k*, the average path length *L*, and the clustering coefficient *C*^16^. The density of the network *D* is defined as the ratio of actual edges to potential edges,

3 $$D{ } = \frac{2E}{{N\left( {N{-}1} \right)}}$$

where *N* is the number of nodes while *E* is the number of edges, factor 2 arises for undirected networks only. The average node degree *k* can be calculated by:

4 $$k = \frac{2E}{N}$$

where factor 2 is for undirected networks again. In WCL, *E* is replaced by the sum of the edge weights. The average path length *L* is the average number of steps along the shortest paths for every pair of nodes:

5 $$L = \frac{2}{{N\left( {N - 1} \right)}}\mathop \sum \limits_{i \ge j} d_{ij}$$

where factor 2 is for undirected networks again, *d*_*ij*_ is the shortest path length between a pair of nodes *i* and *j*. Finally, the clustering coefficient *C* is the measure of the degree to which nodes in a network will cluster together. It can be calculated as:

6 $$C = \frac{1}{N}\mathop \sum \limits_{i = 1}^{N} \frac{{2e_{i} }}{{k_{i} \left( {k_{i} - 1} \right)}}$$

with *e*_*i*_ being the number of edges between the neighbors of node *i*, *k*_*i*_ being the number of neighbors of node *i*. We further performed spline interpolation to analyze the spatial distribution of node degree and betweenness centrality of all nodes in the network.

As an alternative method to network analyses, we performed spatial point pattern analysis^26,28,40,59^ to characterize tree spatial patterns. *L*(*r*) and *g*(*r*) were chosen to describe the second-order point pattern. *L*(*r*) is a transformation of Ripley's *K* function proposed by Besag^60^ while *g*(*r*) is the pair correlation function. Second-order spatial patterns corresponds to aggregated [*g*(*r*) > 1], random [*g*(*r*) = 1], or regular patterns [*g*(*r*) < 1]^25^. Given the null hypothesis of CSR, a total of 199 Monte-Carlo simulations were performed, generating a confidence envelope to describe the maximum and minimum values of simulated results. Spatial point pattern analysis was conducted using the ‘spatstat’ package^28^ in R software, version 4.0.2. Mathematical terms with their ecological interpretations in this study are listed in Supplementary Table S3.

## Supplementary Information

Supplementary Information.

## Data Availability

The datasets used for our case study can be obtained in the study by Ferraz, Antonio, et al. (https://zenodo.org/record/4934622#.YrMODUbP2ix).

## Code availability

All codes used for our simulated study and case study can be found on the GitHub repository (https://github.com/Asa12138/Forest_net).
