# Supplementary material for: Rethinking the complexity and uncertainty of spatial networks applied to forest ecology
Source: Sci Rep. 2022 Sep 23;12:15917. doi: 10.1038/s41598-022-16485-9 (PMC9508254; doi:10.1038/s41598-022-16485-9)
Supplement: Supplementary file 1 — Supplementary Information. [file 41598_2022_16485_MOESM1_ESM.docx]

| Network | Model | *N* | | | *E* | | | *k* | | | *C* | | | *D* | | | *L* | | |
| --- | --- | --- | --- | --- | --- | --- | --- | --- | --- | --- | --- | --- | --- | --- | --- | --- | --- | --- | --- |
|  |  | min | mean | max | min | mean | max | min | mean | max | min | mean | max | min | mean | max | min | mean | max |
| CS | Tho | 469 | 590 | 733 | 1263 | 2240 | 3215 | 5.39 | 7.55 | 9.16 | 0.75 | 0.82 | 0.88 | 0.0111 | 0.0128 | 0.0164 | 1.77 | 3.08 | 6.06 |
|  | Mat | 450 | 590 | 747 | 1392 | 2194 | 3301 | 5.88 | 7.41 | 9.21 | 0.71 | 0.76 | 0.83 | 0.0104 | 0.0126 | 0.0172 | 2.27 | 5.36 | 13.64 |
|  | CSR | 518 | 590 | 666 | 1051 | 1345 | 1712 | 4.00 | 4.55 | 5.15 | 0.55 | 0.59 | 0.64 | 0.0072 | 0.0077 | 0.0084 | 6.72 | 17.90 | 30.77 |
|  | Str | 375 | 432 | 485 | 535 | 701 | 848 | 2.85 | 3.25 | 3.55 | 0.47 | 0.53 | 0.58 | 0.0067 | 0.0075 | 0.0085 | 5.20 | 10.86 | 26.47 |
|  | HC | 290 | 333 | 381 | 335 | 413 | 520 | 2.24 | 2.48 | 2.80 | 0.37 | 0.44 | 0.51 | 0.0068 | 0.0075 | 0.0087 | 2.94 | 6.62 | 16.22 |
| CL | Tho | 471 | 578 | 709 | 1094 | 1532 | 2251 | 4.35 | 5.29 | 6.89 | 0.78 | 0.84 | 0.89 | 0.0075 | 0.0092 | 0.0112 | 1.45 | 2.09 | 3.98 |
|  | Mat | 434 | 580 | 739 | 913 | 1420 | 2124 | 4.05 | 4.88 | 6.03 | 0.66 | 0.72 | 0.77 | 0.0069 | 0.0085 | 0.0102 | 1.92 | 3.38 | 6.50 |
|  | CSR | 463 | 527 | 602 | 554 | 687 | 868 | 2.27 | 2.60 | 2.95 | 0.51 | 0.57 | 0.65 | 0.0043 | 0.0050 | 0.0055 | 2.40 | 3.82 | 7.68 |
|  | Str | 302 | 348 | 411 | 256 | 335 | 446 | 1.62 | 1.92 | 2.29 | 0.36 | 0.47 | 0.57 | 0.0047 | 0.0055 | 0.0066 | 1.84 | 2.62 | 6.09 |
|  | HC | 185 | 231 | 293 | 128 | 177 | 236 | 1.36 | 1.53 | 1.72 | 0.15 | 0.31 | 0.44 | 0.0055 | 0.0067 | 0.0080 | 1.54 | 2.09 | 3.52 |
| WCL | Tho | 449 | 586 | 706 | 1954 | 3148 | 4594 | 17.86 | 21.59 | 26.48 | 0.77 | 0.83 | 0.90 | 0.0077 | 0.0092 | 0.0114 | 3.00 | 4.16 | 6.43 |
|  | Mat | 428 | 575 | 698 | 1878 | 2811 | 3948 | 12.34 | 14.91 | 18.13 | 0.66 | 0.72 | 0.78 | 0.0071 | 0.0085 | 0.0103 | 3.17 | 4.52 | 8.71 |
|  | CSR | 441 | 529 | 608 | 1014 | 1380 | 1704 | 5.77 | 6.86 | 7.88 | 0.50 | 0.57 | 0.63 | 0.0044 | 0.0049 | 0.0058 | 4.14 | 6.42 | 16.16 |
|  | Str | 286 | 349 | 405 | 496 | 675 | 864 | 3.51 | 4.46 | 5.58 | 0.38 | 0.47 | 0.54 | 0.0049 | 0.0055 | 0.0068 | 3.13 | 4.55 | 6.74 |
|  | HC | 172 | 231 | 276 | 266 | 357 | 476 | 2.11 | 2.69 | 3.36 | 0.13 | 0.31 | 0.46 | 0.0058 | 0.0068 | 0.0090 | 2.31 | 3.44 | 6.16 |

**Supplementary Table S1.** Basic characteristics of three types of the networks (Competition for space, CS; Competition for light, CL; weighted competition for light, WCL) based on each spatial null model (Thomas process, Tho; Matérn process, Mat; Complete spatial randomness, CSR; Strass process, Str; Gibbs hard core process, HC). The minimum, averaged, and maximum of the network metrics (the number of nodes *N*, the number of edges *E*, the average node degree *k*, the clustering coefficient *C*, the density of edge *D*, and the average path length *L*) across 199 Monte-Carlo simulations are shown.

|  | Group | CS | | | | CL | | | | WCL | | | |
| --- | --- | --- | --- | --- | --- | --- | --- | --- | --- | --- | --- | --- | --- |
|  |  | *k* | *C* | *D* | *L* | *k* | *C* | *D* | *L* | *k* | *C* | *D* | *L* |
| ANOVA | All | 0 | 0 | 0 | 1.3E-312 | 0 | 0 | 0 | 2.7E-166 | 0 | 0 | 0 | 2.5E-139 |
| Tukey | Mat-Tho | 0.00628 | 4.48E-13 | 0.005 | 5.82E-13 | 4.48E-13 | 4.48E-13 | 4.48E-13 | 4.48E-13 | 4.48E-13 | 4.48E-13 | 4.48E-13 | 0.00555 |
|  | CSR-Tho | 4.48E-13 | 4.48E-13 | 4.48E-13 | 4.48E-13 | 4.48E-13 | 4.48E-13 | 4.48E-13 | 4.48E-13 | 4.48E-13 | 4.48E-13 | 4.48E-13 | 4.48E-13 |
|  | Str-Tho | 4.48E-13 | 4.48E-13 | 4.48E-13 | 4.48E-13 | 4.48E-13 | 4.48E-13 | 4.48E-13 | 5.06E-13 | 4.48E-13 | 4.48E-13 | 4.48E-13 | 0.00149 |
|  | HC-Tho | 4.48E-13 | 4.48E-13 | 4.48E-13 | 4.48E-13 | 4.48E-13 | 4.48E-13 | 4.48E-13 | 1.000 | 4.48E-13 | 4.48E-13 | 4.48E-13 | 3.13E-11 |
|  | CSR-Mat | 4.48E-13 | 4.48E-13 | 4.48E-13 | 4.48E-13 | 4.48E-13 | 4.48E-13 | 4.48E-13 | 1.28E-10 | 4.48E-13 | 4.48E-13 | 4.48E-13 | 4.48E-13 |
|  | Str-Mat | 4.48E-13 | 4.48E-13 | 4.48E-13 | 4.48E-13 | 4.48E-13 | 4.48E-13 | 4.48E-13 | 4.49E-13 | 4.48E-13 | 4.48E-13 | 4.48E-13 | 0.997 |
|  | HC-Mat | 4.48E-13 | 4.48E-13 | 4.48E-13 | 0.000121 | 4.48E-13 | 4.48E-13 | 4.48E-13 | 4.48E-13 | 4.48E-13 | 4.48E-13 | 4.48E-13 | 4.95E-13 |
|  | Str-CSR | 4.48E-13 | 4.48E-13 | 0.0328 | 4.48E-13 | 4.48E-13 | 4.48E-13 | 4.49E-13 | 4.48E-13 | 4.48E-13 | 4.48E-13 | 4.49E-13 | 4.48E-13 |
|  | HC-CSR | 4.48E-13 | 4.48E-13 | 0.00161 | 4.48E-13 | 4.48E-13 | 4.48E-13 | 4.48E-13 | 4.48E-13 | 4.48E-13 | 4.48E-13 | 4.48E-13 | 4.48E-13 |
|  | HC-Str | 4.48E-13 | 4.48E-13 | 0.901 | 4.48E-13 | 4.48E-13 | 4.48E-13 | 4.48E-13 | 5.06E-13 | 4.48E-13 | 4.48E-13 | 4.48E-13 | 4.87E-13 |

**Supplementary Table S2.** *p*-values of ANOVA and Tukey tests of difference of network-based metrics (the average node degree *k*, the clustering coefficient *C*, the density *D*, and the average path length *L*) of each network (Competition for space, CS; Competition for light, CL; weighted competition for light, WCL) between different spatial null models (Thomas process, Tho; Matérn process, Mat; Complete spatial randomness, CSR; Strass process, Str; Gibbs hard core process, HC). The *p*-values of each comparison in the Tukey test are adjusted by the Bonferroni method. The red color indicates no significance.

| **term** | **abbreviation/**  **symbol** | **definition** | **ecological interpretation/assumption** |
| --- | --- | --- | --- |
| complete spatial randomness | CSR | spatial null model where the number of points in any region follows a Poisson distribution. Points are independent to each other. | ecological processes are random |
| cluster process | / | a type of spatial null models that generate aggregated patterns. It first generates parent points, and then each gives rise to a Poisson number of offspring, independently distributed in a circular area centered at the parent. | diserpsal limitation |
| Matérn process | Mat | spatial null model which belongs to cluster process. Offspring are uniformly distributed around the parent within a distance. | diserpsal limitation |
| Thomas process | Tho | spatial null model which belongs to cluster process. The number of offspring decreases with increasing distance from parents and has a Gaussian distribution. | diserpsal limitation |
| Gibbs process | / | a type of spatial null models that generate regular patterns. It is simulated by trial and error. Based on a suitable intial pattern, points are removed at random and replaced only by points that are likely to occur given the underlying rules of the process. | competition exclusion |
| Gibbs hard core process | HC | spatial null model which belongs to Gibbs process. Only points that are not close than the hard core distance from any other points will be retained. | competition exclusion |
| Strauss proecss | Str | spatial null model which belongs to Gibbs process. Points that are close than the hard core distance from another point will be removed at a certain probability. | competition exclusion |
| density | D | network metric. The ratio of actual edges to potential edges in the network. | / |
| average node degree | k | network metric. The number of edges that a point have onaverage in the network. | / |
| average path length | L | network metric. The average number of steps along the shortest paths for every pairs of nodes in the network. | / |
| clustering coefficient | C | network metric. The degree to which nodes in a network will cluster together. | / |
| betweenness centrality | / | the number of shortest paths going through a node | / |
| competition for space | CS | network definition where trees were connected if they are closed to each other | trees competes for space |
| competition for light | CL | network definition where trees were connected only if they their crowns are overlapped | trees competes for light |
| weighted competition for light | WCL | network definition where trees were connected only if they their crowns are overlapped. Each interaction is assigned with intensity value indicating competition strength. | trees competes for light with different intensity |

**Supplementary Table S3.** Glossary of spatial null models, network metrics, and other mathematical definitions used in the article.


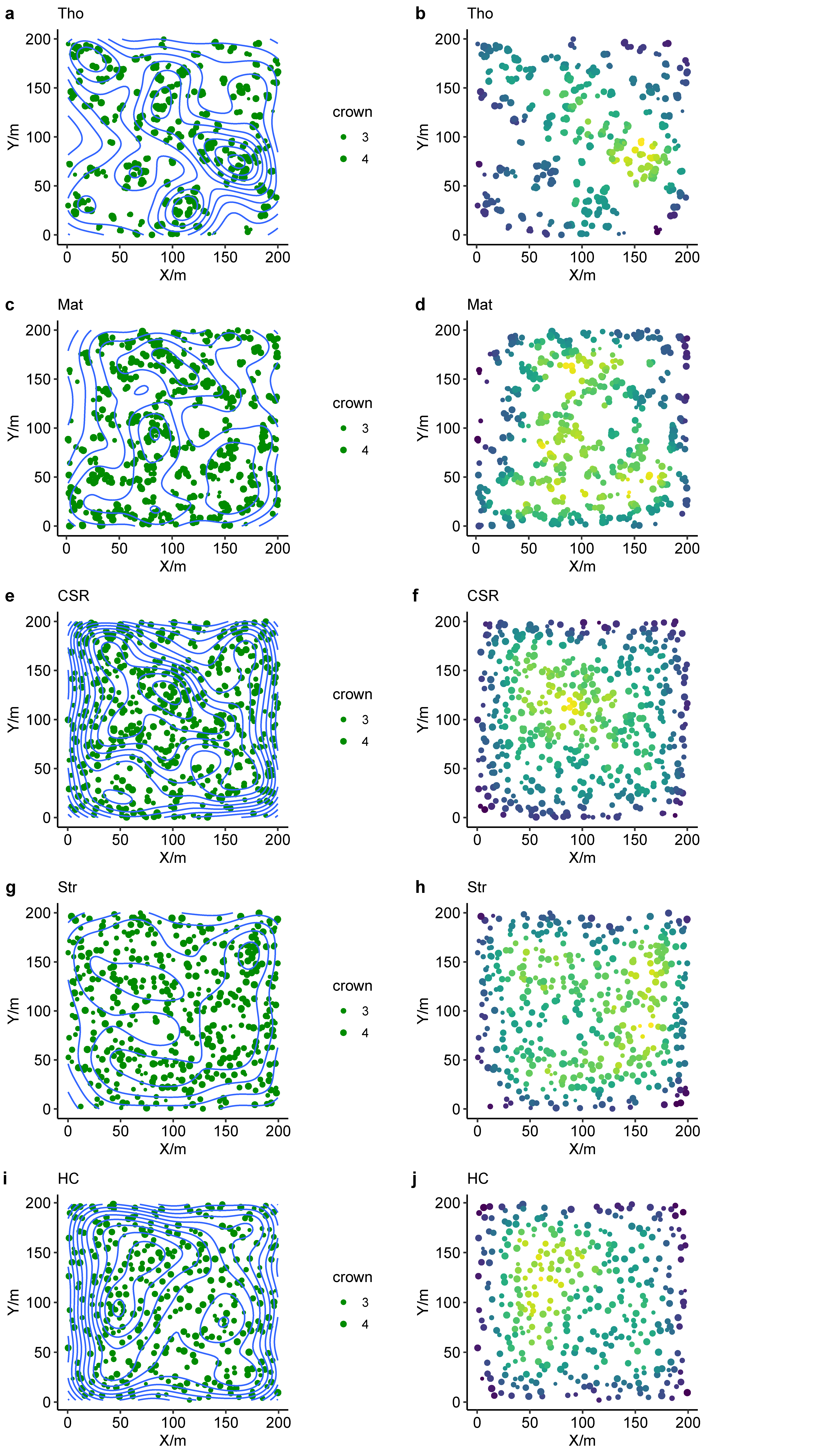


**Supplementary Figure S1.** Distribution of trees in each spatial null model (Thomas process, Tho; Matérn process, Mat; Complete spatial randomness, CSR; Strass process, Str; Gibbs hard core process, HC). (a, c, e, g, i) The blue contour indicates the density of trees; (b, d, f, h, j) Light colors indicate a high density of points.


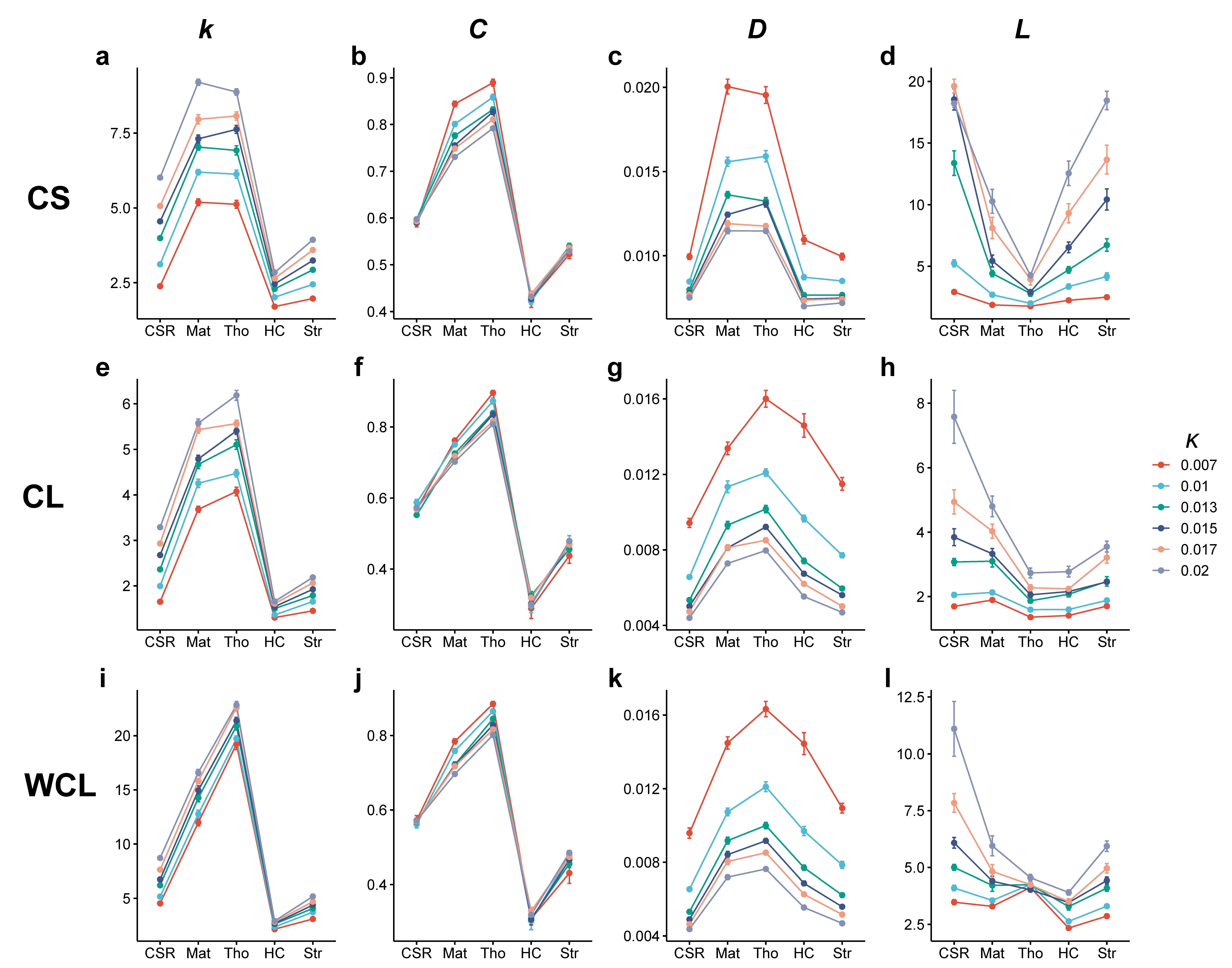
 **Supplementary Figure S2.** Sensitivity test of the point intensity *κ* on network metrics (the average node degree *k*, the clustering coefficient *C*, the density *D*, and the average path length *L*) in three networks (Competition for space, CS; Competition for light, CL; weighted competition for light, WCL) based on five spatial null models (Thomas process, Tho; Matérn process, Mat; Complete spatial randomness, CSR; Strass process, Str; Gibbs hard core process, HC). Each point is the value averaged over 199 Monte-Carlo simulations. The error bar represents the standard deviation.


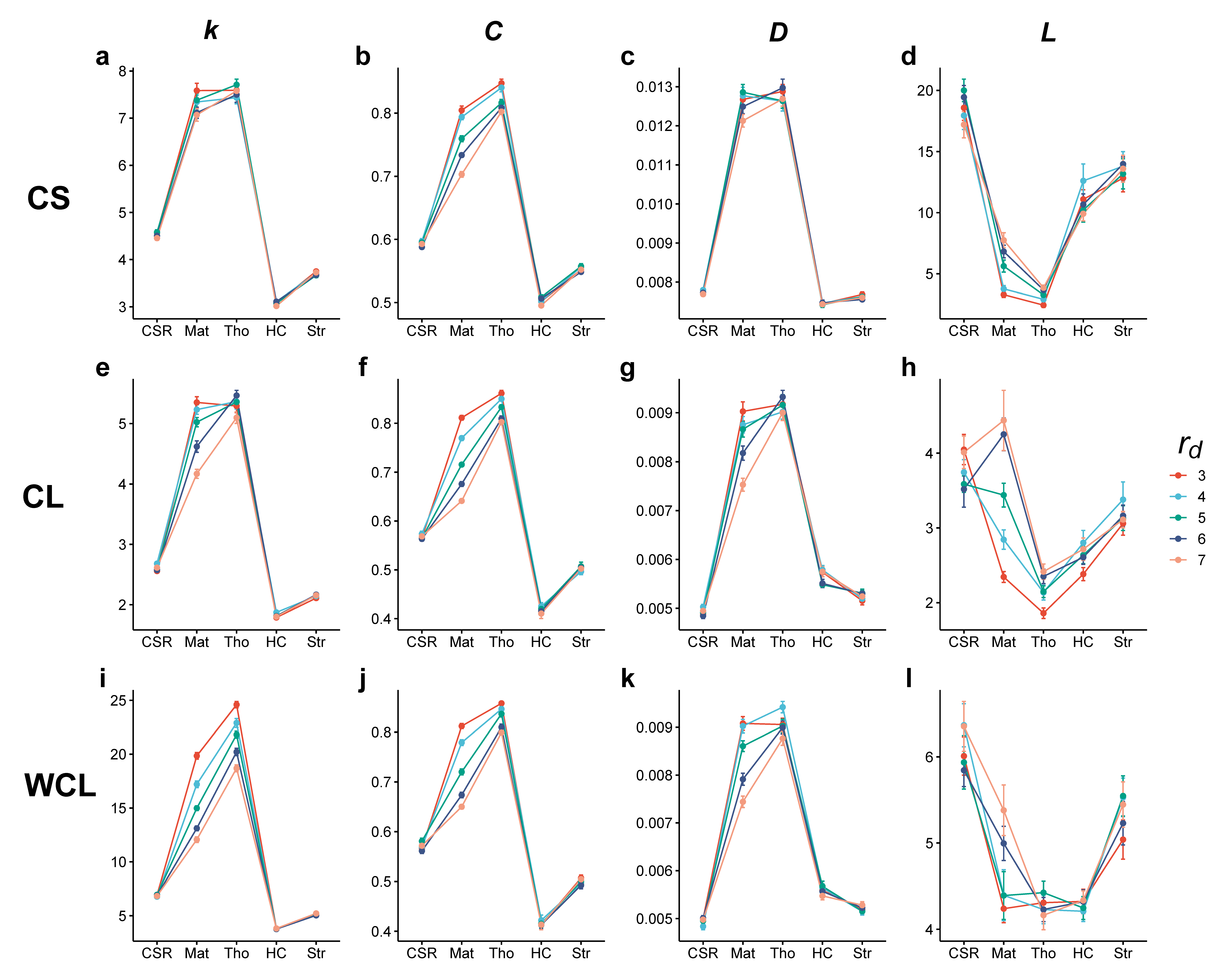


**Supplementary Figure S3.** Sensitivity test of the radius of the dispersal area *r_d_* on network metrics (the average node degree *k*, the clustering coefficient *C*, the density *D*, and the average path length *L*) in three networks (Competition for space, CS; Competition for light, CL; weighted competition for light, WCL) based on five spatial null models (Thomas process, Tho; Matérn process, Mat; Complete spatial randomness, CSR; Strass process, Str; Gibbs hard core process, HC). Each point is the value averaged over 199 Monte-Carlo simulations. The error bar represents the standard deviation.


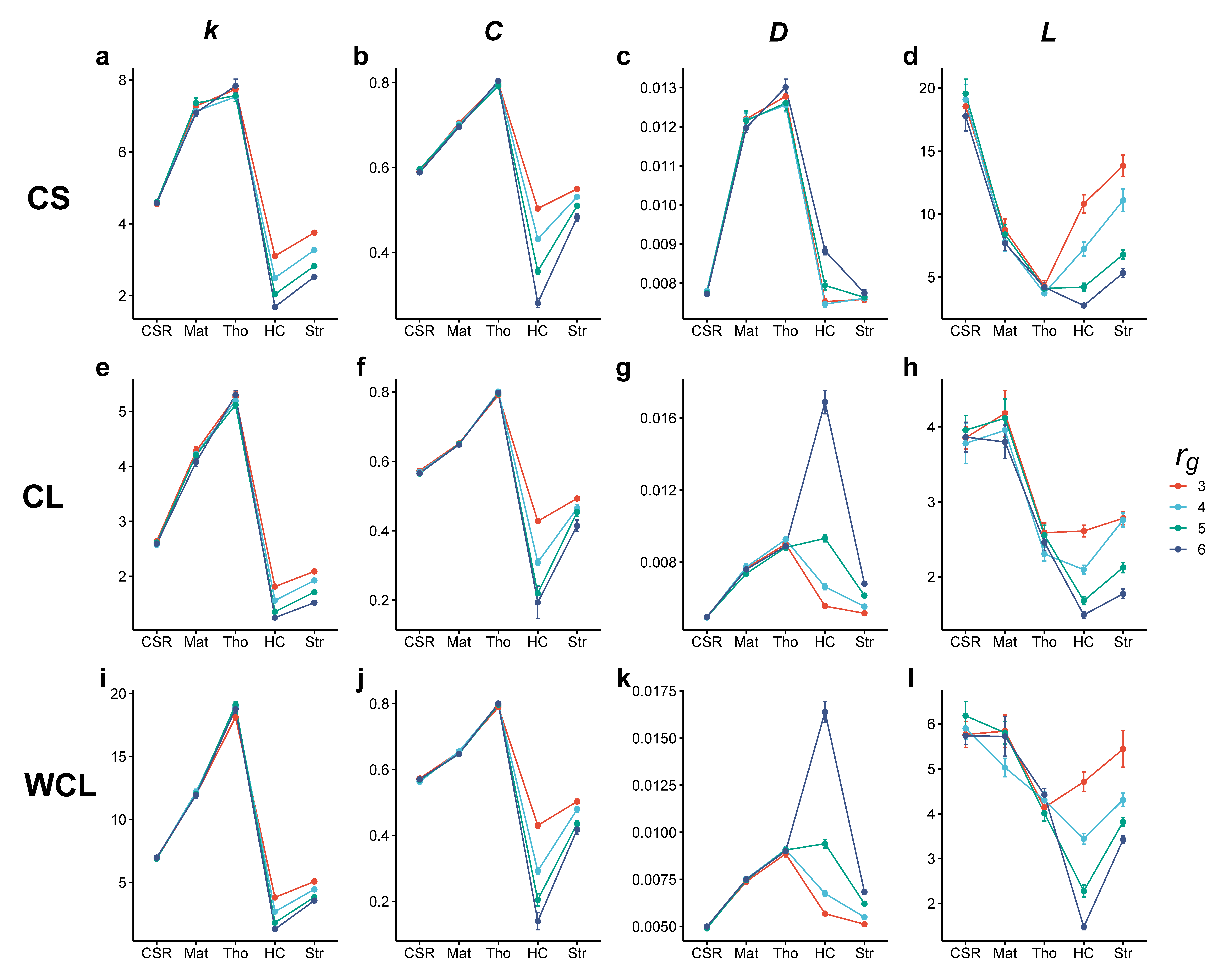


**Supplementary Figure S4.** Sensitivity test of the hard core distance *r_g_* on network metrics (the average node degree *k*, the clustering coefficient *C*, the density *D*, and the average path length *L*) in three networks (Competition for space, CS; Competition for light, CL; weighted competition for light, WCL) based on five spatial null models (Thomas process, Tho; Matérn process, Mat; Complete spatial randomness, CSR; Strass process, Str; Gibbs hard core process, HC). Each point is the value averaged over 199 Monte-Carlo simulations. The error bar represents the standard deviation.


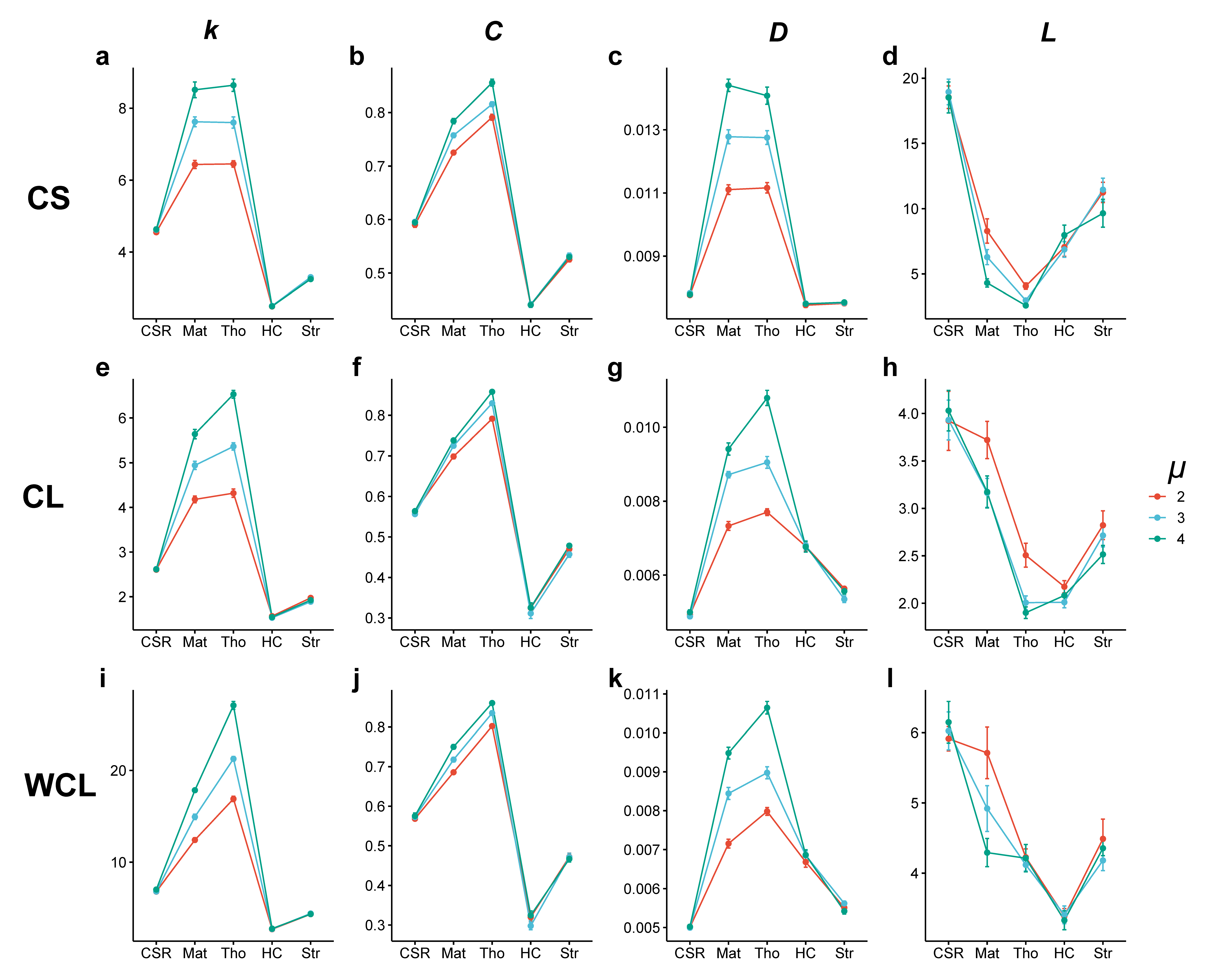


**Supplementary Figure S5.** Sensitivity test of the average number of the offspring per parent *μ* on network metrics (the average node degree *k*, the clustering coefficient *C*, the density *D*, and the average path length *L*) in three networks (Competition for space, CS; Competition for light, CL; weighted competition for light, WCL) based on five spatial null models (Thomas process, Tho; Matérn process, Mat; Complete spatial randomness, CSR; Strass process, Str; Gibbs hard core process, HC). Each point is the value averaged over 199 Monte-Carlo simulations. The error bar represents the standard deviation.


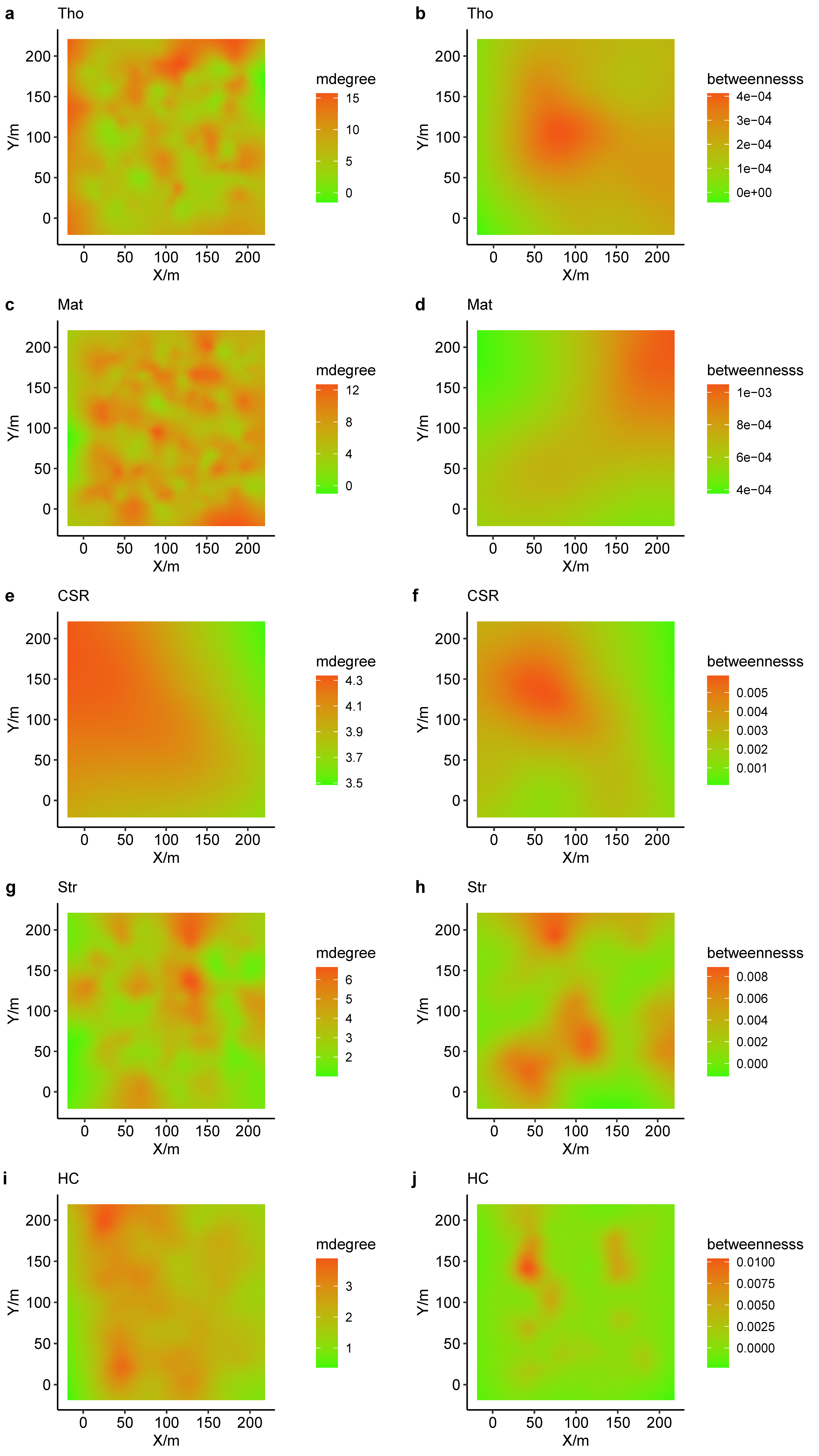


**Supplementary Figure S6.** Distribution of the node attributes based on spline interpolation in the CS network. The deep color indicates high values. (a, c, e, g, i) Node degree; (b, d, f, h, j) Normalized betweenness centrality. (a, b) Thomas process; (c, d) Matérn process; (e, f) Complete spatial randomness; (g, h) Strauss process; (i, j) Gibbs hard core process).


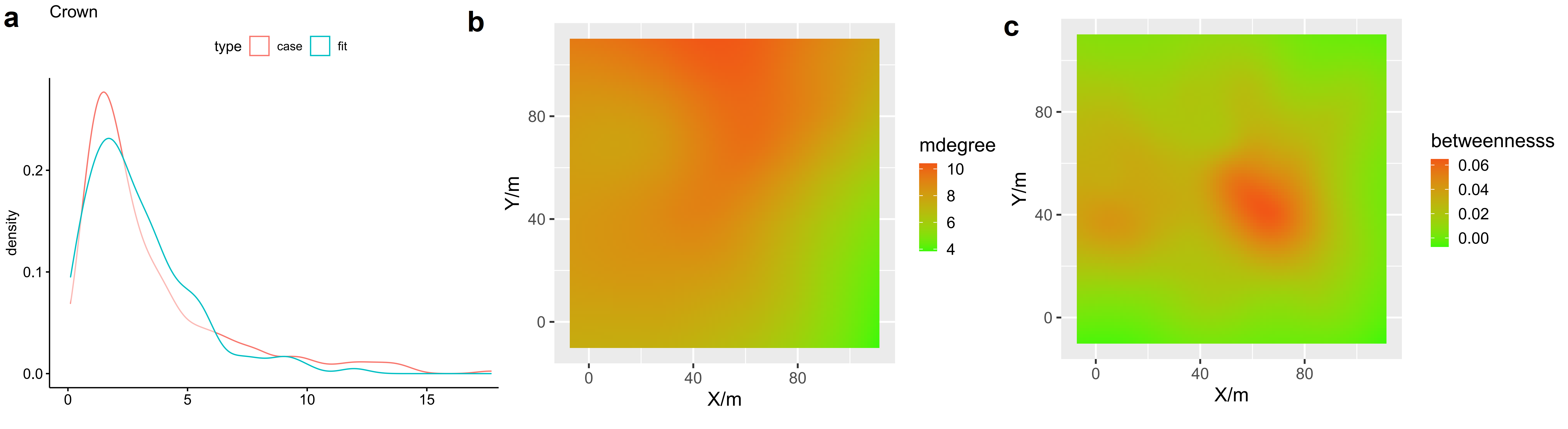


**Supplementary Figure S7.** (a) Distribution of the crown radius in the forested swamp plot FS-1 in Costa Rica. The red curve indicates the observed values while fitted values are presented by the green curve; (b, c) Distribution of node attributes based on spline interpolation in the CS network for the forest dataset. The deep color indicates high values.
